# Disulfide radical anion as a super-reductant in biology and photoredox chemistry†

**DOI:** 10.1039/d3sc01867a

**Published:** 2023-05-17

**Authors:** Qilei Zhu, Cyrille Costentin, JoAnne Stubbe, Daniel G. Nocera

**Affiliations:** a Department of Chemistry and Chemical Biology, Harvard University 12 Oxford Street Cambridge Massachusetts 02138 USA dnocera@fas.harvard.edu; b Department of Chemistry, University of Utah Salt Lake City Utah 84112 USA; c Université Grenoble Alpes, CNRS, DCM 38000 Grenoble France; d Departments of Chemistry and Department of Biology, Massachusetts Institute of Technology Cambridge Massachusetts 02139 USA

## Abstract

Disulfides are involved in a broad range of radical-based synthetic organic and biochemical transformations. In particular, the reduction of a disulfide to the corresponding radical anion, followed by S–S bond cleavage to yield a thiyl radical and a thiolate anion plays critical roles in radical-based photoredox transformations and the disulfide radical anion in conjunction with a proton donor, mediates the enzymatic synthesis of deoxynucleotides from nucleotides within the active site of the enzyme, ribonucleotide reductase (RNR). To gain fundamental thermodynamic insight into these reactions, we have performed experimental measurements to furnish the transfer coefficient from which the standard *E*^0^(RSSR/RSSR˙^−^) reduction potential has been determined for a homologous series of disulfides. The electrochemical potentials are found to be strongly dependent on the structures and electronic properties of the substituents of the disulfides. In the case of cysteine, a standard potential of *E*^0^(RSSR/RSSR˙^−^) = −1.38 V *vs.* NHE is determined, making the disulfide radical anion of cysteine one of the most reducing cofactors in biology.

## Abstract 2

Peak and half-peak potentials poorly approximate standard reduction potentials (*E*^0^), especially for compounds that dissociate upon reduction. We determine *E*^0^ of disulfides and show cysteine disulfide radical anion to be a powerful biological reductant.

## Graphical abstract

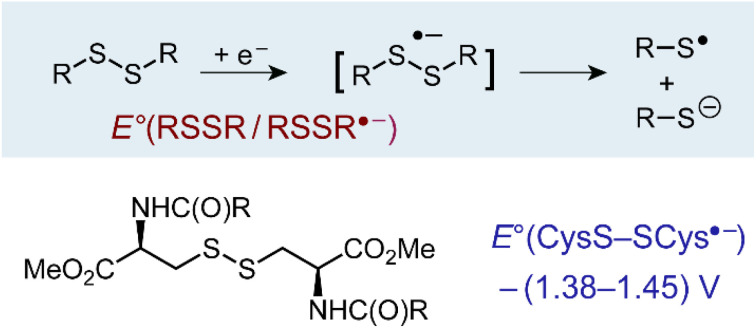

## Introduction

**Fig. 1:**
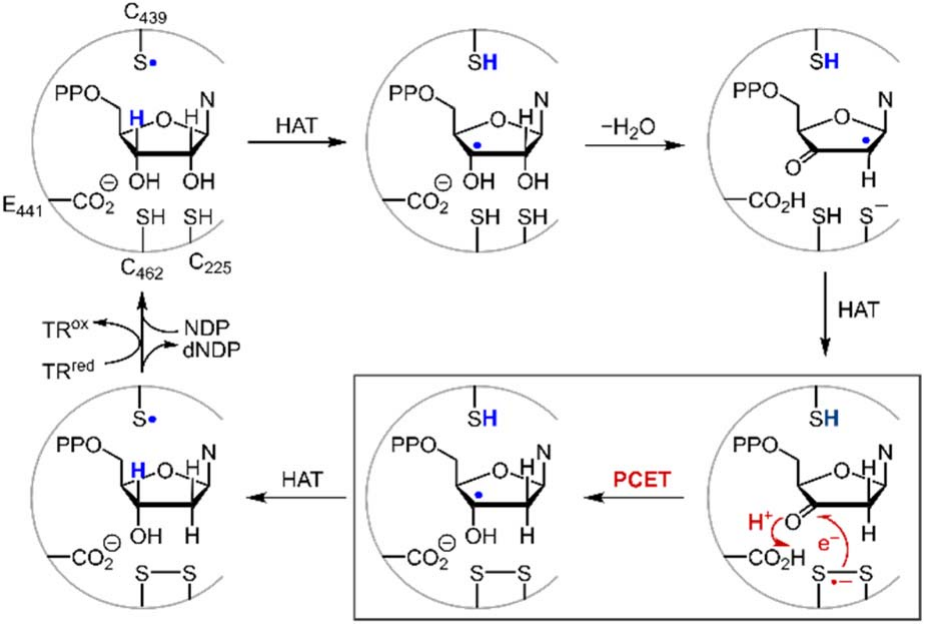
Proposed mechanism for the biosynthesis of deoxynucleotides within the active site of class Ia ribonucleotide reductase.

Disulfide redox couples are central to many radical-mediated organic and biochemical reactions.^1,2^ With the emergence of photoredox catalysis, disulfides are intermediates^3–5^ in promoting versatile regio- and stereo-selective hydrofunctionalizations of unactivated olefins. Outside of the flask, nature exploits the unique redox properties of the disulfide group derived from cysteine in a broad range of critical biochemical processes.^6–8^ Indeed, the universal and essential process of the reduction of the four nucleotides to the four deoxynucleotides for DNA replication and repair is driven by the radical chemistry of thiol and disulfide.^9,10^Fig. 1 shows the proposed mechanism for the production of the deoxynucleotide building blocks by the class Ia ribonucleotide reductase (RNR) enzymes.^11,12^ The disulfide arises from the oxidation of the two bottom face cysteines. The formation of the disulfide radical anion results from loss of a molecule of water to generate a radical (top right, Fig. 1), which then is reduced by H-atom transfer from the bottom face cysteine to form the 3′-ketone deoxynucleotide and the radical anion (bottom right, Fig. 1). The disulfide radical anion then reacts by the PCET pathway shown by the red arrows in Fig. 1; a proton supplied by the carboxylate and an electron supplied by the disulfide radical anion results in reduction of the ketone. The 3′-H removed from the NDP substrate by the top face thiol is then returned to the same position in the product (blue H). When the carboxylic acid is removed as the proton source, the disulfide radical anion is spectroscopically detected^13^ and has been invoked as a key intermediate in the reduction of 2′-deoxy-3′-ketone (box in Fig. 1). Notwithstanding, beyond RNR, the disulfide radical anion has largely been overlooked as a reductant in biology owing to its fleeting stability. The LUMO of disulfide has major contributions from the σ*_S–S_ anti-bonding orbital^14^ and hence S–S bond cleavage is facile upon reduction. Indeed, the rate constants of S–S dissociation of several RSSR˙^−^ species have been measured to be as large as 10^5^–10^6^ s^−1^.^15,16^

**Scheme 1:**
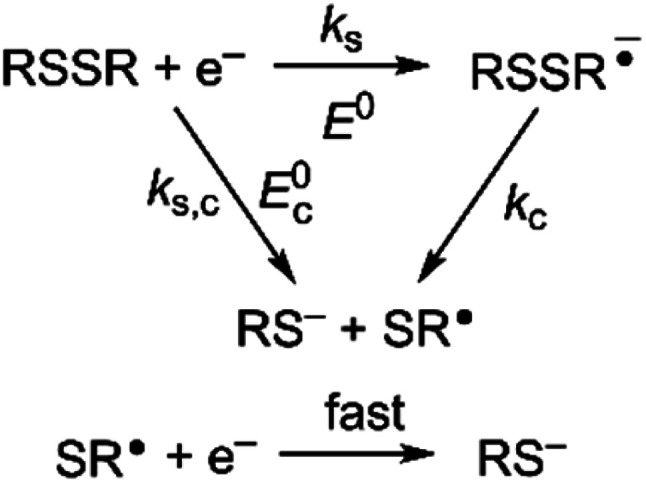
Concerted and stepwise pathways for dissociative RSSR reduction by two electrons to yield thiolate.

Owing to its instability, the thermodynamic values of most disulfides are ill-defined for accurate determination of *E*^0^. Standard reduction potentials of RSSR/RSSR˙^−^ redox couples have been computed with knowledge of bond dissociation energies and p*K*_a_ values.^17^ However, expanding this method to a broad range of disulfides is challenging due to the lack of readily available thermodynamic data. The preferred approach of using cyclic voltammetry to ascertain the standard reduction potential is convoluted by the facile S–S bond-cleavage, which gives rise to an irreversible wave in the CV due to a stepwise ECE (E = electron transfer, C = chemistry) process or alternatively an initial concerted dissociative electron transfer followed by a fast reduction of the ensuing radical (Scheme 1).^18^ For irreversible redox processes, the peak potential is often chosen as an approximation of the reduction potential. However, such an approximation is problematic for the reduction of disulfides. In the case of a stepwise pathway, *E*_p_ depends on the rate constant of electron transfer, *k*_S_, in the framework of a Butler–Volmer kinetic law,1
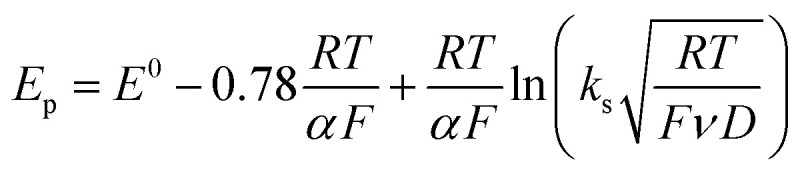
where *D* is the diffusion coefficient. Reductive cleavage of disulfides has been shown to be kinetically controlled by the initial electron transfer.^19^ In this case, a small value of *k*_s_ results in an *E*_p_ that is more negative than *E*^0^. The same form of the relation between the peak and standard potential is obtained for the concerted dissociative electron transfer pathway of Scheme 1. In this case, the rate determining electron transfer step is convoluted with chemical bond breaking, and the rate constant, *k*_s,c_, is even smaller owing to a large reorganization energy. Here, the peak potential will be much more negative than the corresponding dissociative standard potential *E*^0^_c_,2
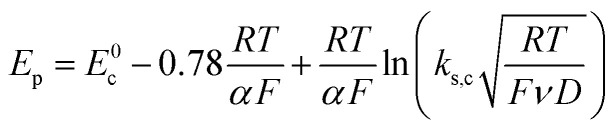


Hence, whether stepwise or concerted, large excursions in *E*_p_ relative to the standard potential, *E*^0^ (or *E*^0^_c_) should be expected for the reduction of disulfides.

While *E*_p_ does not accurately reflect the standard potential for disulfide reduction, the standard potential may be determined from *E*_p_ with knowledge of the transfer coefficient, α,^20^ which is a unitless value that describes the dependence of activation free energy on thermodynamic driving force of electron transfer.^21,22^ Using this formalism, we now report the standard redox potential of a diverse series of disulfides, including that of cysteine. We show that the substitution of the disulfide moiety engenders a large range in *E*^0^ that spans nearly 1.0 V. Importantly, the standard *E*^0^(RSSR˙^−^/RSSR) potential is poorly approximated by *E*_p_ and moreover, the relative values of *E*_p_ among RSSR derivatives do not scale with *E*^0^. Measurement of the cysteine disulfide shows it to be a strong reducing cofactor, with a *E*^0^(RSSR˙^−^/RSSR) = −1.38 V *vs.* NHE, thus engendering it as a biological super-reductant. The CysS–SCys˙^−^ is so reducing that it is in the range of the reduction of cyclic ketones, the potentials of which have been determined from a Breslow–Bordwell thermodynamic cycle. These standard potentials are consistent with the active site chemistry of ribonucleotide reductase where CysS–SCys˙^−^ is harnessed to drive the PCET conversion of nucleotides to deoxynucleotides, thus underpinning the redox process that supplies the building blocks for DNA synthesis and repair.

## Results and discussion

**Fig. 2:**
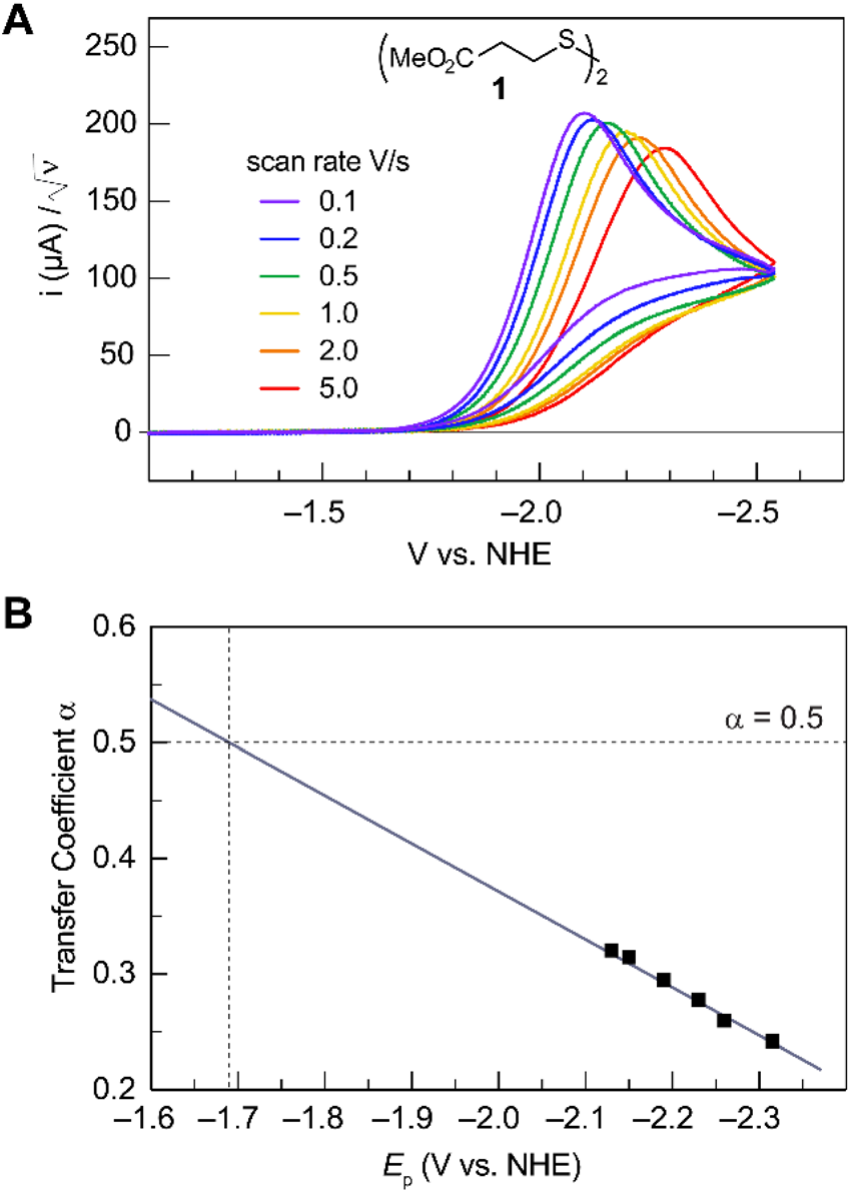
(A) Cyclic voltammogram of disulfide 1. Electrolyte is anhydrous acetonitrile solution containing 1.5 mM RSSR and 0.1 M tetrabutylammonium hexafluorophosphate. (B) Peak potential (*E*_p_)-transfer coefficient (*α*) plot of disulfide 1.

Fig. 2A shows the cyclic voltammogram for the reduction of dialkyl disulfide substrate 1 at scan rates of 0.1 V s^−1^ to 5.0 V s^−1^. A glassy carbon working electrode was chosen to avoid specific interaction between the electrode surface and disulfides and thus enforce an outer-sphere electron transfer reaction. An irreversible two-electron wave is observed as a result of the two-electron mechanism shown in Scheme 1. The complete irreversibility is due to the initial EC process with the rate of the chemical step far exceeding the time duration of the cyclic voltammogram set by the scan rate.^23,24^ The gradual cathodic shift of the peak potentials (110 mV/log *v*) with increasing scan rates is consistent with previous observations of dissociative electron transfer in aromatic disulfides. More suitable than the empirical Butler–Volmer kinetic law, the activation free energy, Δ*G*^‡^, in the Marcus formalism of outer-sphere ET is,^25,26^3
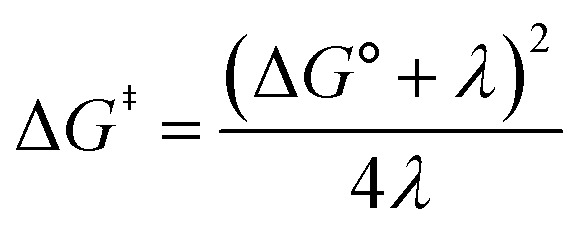
Formally, the transfer coefficient is defined as,4
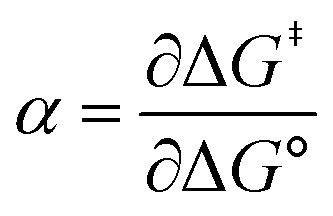
and therefore,5
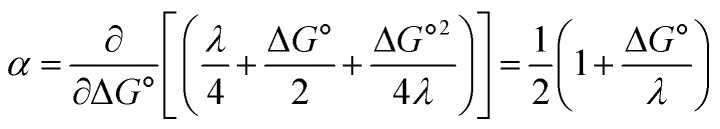


At Δ*G*° = 0, *α* = 0.5, which is the condition where *E* = *E*^0^. Thus, a plot of *α vs. E*_p_ yields *E*^0^(RSSR˙^−^/RSSR) at *α* = 0.5 and the slope of the plot gives the reorganization energy *λ*, provided that the *α vs. E*_p_ plot is linear. An average value of *α* can be determined from the peak potential (*E*_p_) as a function of scan rate, *ν*,^18^6
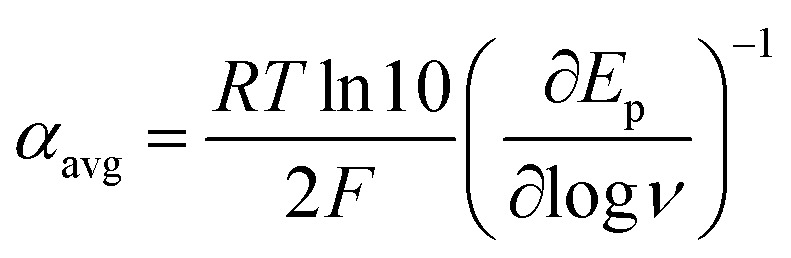


The variation of *α* with the driving force can be more precisely obtained from the peak width of CVs at each scan rate, which is the potential difference at the mid-peak height *E*_p/2_,7
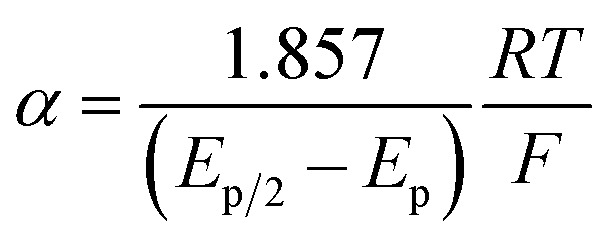


**Fig. 3:**
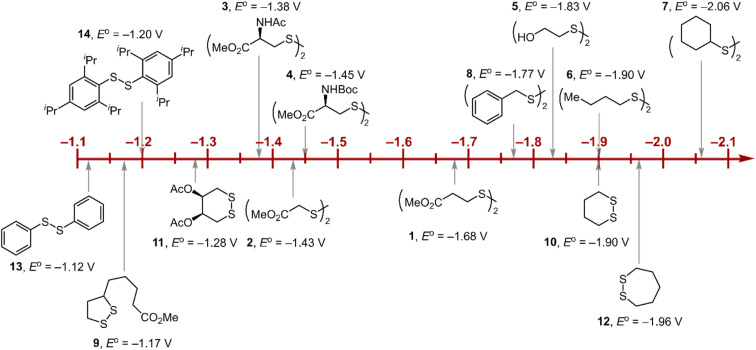
Standard reduction potentials of alkyl and aromatic disulfides. Compounds 3 and 13 have been previously reported in ref. 24 and 20, respectively.

Within the formalisms defined by eqn (6) and (7), the cyclic voltammetry of the series of disulfides shown in Fig. 3 has been evaluated. Besides the commercially available disulfides, 1, 3, 5–8 and previously prepared 14;^3^ the preparation and characterization of compounds 2, 4, 9–12 are presented in ESI Section B.† At scan rates from 0.1 to 5 V s^−1^, transfer coefficients of disulfide 1 range from 0.33 to 0.24, indicating the heterogeneous electron transfer is the kinetically rate-limiting step. Fig. 2B shows the *α*–*E*_p_ plot; extrapolation to *α* = 0.5 yields the standard reduction potential, *E*^0^ (1/1˙^−^) = −1.68 V *vs.* NHE. The same analysis was applied to determine the transfer coefficients and standard reduction potentials of the alkyl disulfides (2–12) and aromatic disulfide (14) shown in Fig. 3. Cyclic voltammograms recorded at different scan rates and *α vs. E*_p_ plots are shown in Fig. S1A and B,† respectively. Similar to disulfide 1, transfer coefficients of all disulfides are smaller than 0.5, indicating electron transfer to be accompanied by a large reorganization as revealed from the slopes *λ* evaluated from the slopes of *α vs. E*_p_ plots. The reorganization energy is consistent with a contribution of the bond dissociation energy accompanying electron transfer^27,28^ as indicated by Fig. S2,† which shows that the driving force for the initial electron transfer at the peak potential (*E*_p_ − *E*^0^) correlates well with the reorganization energy.

As highlighted in Fig. 3, the disulfide radical anion constitutes a versatile class of reductants, with standard potentials spanning 1.0 V of reducing power as a result of a significant inductive effect. Reduction potentials of disulfides with esters (1–4), amides (3), carbamates (4), and alcohols (5) generally exhibit anodic shifts compared to simple dialkyl disulfides (6, 7), presumably due to stabilization of the radical anion by the electron-withdrawing groups. Table 1 summarizes the electrochemical data for compounds 1–14 from which important observations emerge from these data.

**Table 1:** Transfer coefficients and standard reduction potentials of disulfides

RSSR^a^	*E*_p_ b ^,^ c/V	*α* b ^,^ d	*E* ^0^ e/V	*E* ^0^–*E*_p_	*λ* h/eV
1	−2.13	0.27	−1.68 ± 0.05	0.45	1.20
2	−1.81	0.24	−1.43 ± 0.01	0.38	1.16
3^f^	−1.84	0.20	−1.38 ± 0.05	0.46	1.33
4	−1.94	0.20	−1.45 ± 0.04	0.49	1.09
5	−1.95	0.21	−1.83 ± 0.02	0.12	0.77
6	−2.32	0.22	−1.90 ± 0.05	0.42	1.21
7	−2.37	0.28	−2.06 ± 0.01	0.31	0.95
8	−2.09	0.22	−1.77 ± 0.01	0.32	1.02
9	−1.84	0.21	−1.17 ± 0.05	0.67	1.53
10	−2.18	0.26	−1.90 ± 0.02	0.28	1.00
11	−1.92	0.21	−1.28 ± 0.08	0.67	1.53
12	−2.30	0.25	−1.96 ± 0.06	0.34	1.11
13^g^	−1.40	0.33	−1.12	0.28	1.10
14	−1.53	0.23	−1.20 ± 0.02	0.33	1.06

a Compounds shown in Fig. 3.

b Cyclic voltammograms recorded on anhydrous acetonitrile solutions containing 1.5 mM RSSR and 0.1 M tetrabutylammonium hexafluorophosphate (Fig. S1A).

c Cathodic peak potential *vs.* NHE.

d Calculated from eqn (7).

e *E* ^0^ determined from extrapolation of *E*_p_–*α* plots to *α* = 0.5 (Fig. S1B).

f Data for compound 3 taken from ref. 24.

g Data for compound 13 taken from ref. 20.

h *λ* determined from the slope of of *E*_p_–*α* plots.

Firstly, peak potentials poorly approximate the standard reduction potential as a result of a small electron transfer rate constant (*k*_s_ in eqn (1)). Moreover, within the series of disulfide compounds, *E*_p_ does not scale relative to *E*^0^. The relative difference between *E*_p_ and *E*^0^ (*i.e.* Δ(*E*_p_ − *E*^0^)) varies by 550 mV. This is a direct result of the varying rate constant for electron transfer and disparate S–S bond dissociation energies.^27^ For instance, compound 9 shows the greatest divergence of *E*_p_ from *E*_0_, *E*_p_–*E*^0^ = 0.67 V. Small ring cyclic disulfides, with ∠C–S–S–C dihedral angles deviating from 90°, are stereoelectronically destabilized due to conformationally disfavored anomeric effects and inefficient orbital overlap.^29^ Hence the rate constant for formation of RSSR˙^−^ in 9 is significantly decreased due to a large reorganization energy driven by the release of ring strain upon reduction. Thus, *E*_p_ is a particularly poor approximations of *E*^0^, and within the series, the values are the most divergent. Even in the absence of ring strain, a consistent value of *E*_p_ relative to *E*^0^ is not observed (Δ(*E*_p_ − *E*^0^) = 370 mV), which indicates that the rate of electron transfer in the absence of ring strain varies significantly with *R* as represented by the variation of the reorganization energies *λ* (Table 1). Thus, not only is *E*_p_ a poor approximation of *E*^0^ but *E*_p_ additionally does not track *E*^0^ in a relative manner among the disulfide homologs.

### Relevance to photoredox chemistry

The disparity between of *E*_p_ from *E*^0^ has significant consequences to the area of photoredox chemistry because it has been advocated that half-peak potentials (*E*_p/2_) are preferred for estimating standard half-cell potentials, *E*^0^, and are a reliable measure for determining Δ*G*^0^.^30^*E*_p_ parallels *E*^0^ only if the reorganization energy remains constant in a series of compounds. However, this is not the case for most substrates, as exemplified here for disulfide compounds. If *E*_p_ is a poor measure of *E*^0^, then *E*_p/2_ is equally a poor measure of *E*^0^ for a given substrate (per eqn (7)). For example, photocatalytic hydroamination reactions have been shown to be driven by the reduction of disulfide 14 with a photogenerated Ir(ii) intermediate.^3^ The reduction is thermodynamically disfavored by 230 mV based on the *E*_p/2_ of 14 (*E*^0^(IrA^III/II^) = −1.17 V, *E*_p/2_(14) = −1.40 V). However, from the *E*^0^(14) = −1.20 V (Table 1), the reaction is actually thermo-neutral. We note that in photoredox transformations, the C step—the chemical reaction of the critical reactive intermediate is often a bimolecular process as opposed to a unimolecular process. Nonetheless, the complete irreversibility of a CV wave is a result of a facile reaction of substrate radical anion/cation (usually with solvent) that likely occurs under pseudo first order conditions. As has been shown,^31^ the peak potential for a fast bimolecular EC process is more positive (for a reduction) or more negative (for an oxidation) than the standard potential. The results reported here highlight that it is imperative to have knowledge of *k*_s_ (*k*_s,c_) if *E*_p_ (and *E*_p/2_) is to be used to approximate *E*^0^.

### Relevance to biological chemistry

A second insight garnered from Table 1 is the reduction potential of *E*^0^(3) = −1.38 V (and *E*^0^(4) = −1.45 V *vs.* NHE) places CysS–SCys˙^−^ as one of the most potent reductants in biology. This disulfide anion is nearly 1 V more reducing than the NADP/NADPH reduction couple and, excluding the elements of the 1st and 2nd columns, it is more reducing than most metals of the periodic table. Such reducing power is essential to the ND(T)P to dND(T)P chemistry of RNR. To evaluate the critical reduction of the furanone intermediate shown in Fig. 1, we performed electrochemical studies of ketone reduction using the 2-acetic acid (15) and ester (16) of 2-cyclohexanone and 2-furanone (17) (Fig. S3†). Propylene carbonate was chosen as the solvent for cyclic voltammetry experiments because of its extended window on the cathodic end. Notwithstanding, direct reduction of 16 and 17 occurs at the solvent edge (Fig. S3†). For the case of 15, a considerable anodic shift of the reduction is observed with an onset potential near −1.5 V (Fig. S3†), revealing the critical role of the carboxylic acid as the proton donor in the proposed concerted PCET reduction mechanism. Analogously, a positive shift in an onset potential was observed when 17 is reduced in the presence of acetic acid. Consistent with the role of a proton donor in shifting the furanone potential, the *E*_441_ proton donor residue (Fig. 1) on the bottom face of the RNR active site appears to be essential for the reduction of the furanone (Fig. 1).^12,32^ However, the *E*^0^ reduction potential of furanone in the presence of protons cannot be determined in the same manner because of the underlying hydrogen evolution current resulting from electrochemical reduction of the carboxylic acid. Fortunately, reduction of furanone to the corresponding ketyl radical may be estimated thermochemically (Fig. S4†) by using the Breslow–Bordwell equation,^33–35^

8 BDFE = 1.37 p*K*_a_ + 23.06*E*^0^ + *C*_G_

where the p*K*_a_ is that of the proton donor, BDFE is the O–H bond dissociation energy of the ketyl radical and *C*_G_ is the free energy for one electron reduction of protons to H˙. The O–H bond dissociation energy of the ketyl radical has been calculated to be 28 kcal mol^−1^ for cyclopentanone,^36^ and the p*K*_a_ for glutamate is 4.45.^37^ Using these values, the reduction potential for furanone is −1.34 V *vs.* NHE. Such a reducing potential is attained by CysS–SCys˙^−^, thus supporting the feasibility of reducing the furanone intermediate by the disulfide radical anion of C_225_ and C_462_ within the active site of RNR. Consistent with the thermodynamic values determined herein, recent pulse radiolysis results show that the cysteine disulfide radical anion is able to reduce model ketone substrates under aqueous conditions.^38^

## Conclusions

The peak potential is generally a poor approximation of standard reduction potential. If *E*_p_ is used to approximate *E*^0^, then it should be established that the reorganization energy remains constant among the series of compounds. Alternatively, as we show here, knowledge of the transfer coefficient and its relation to reorganization energy allows the use of *E*_p_ to extract a more accurate evaluation of *E*^0^.

The thiol-disulfide redox couple, from which many versatile chemical and biological transformations are derived, is exemplary of the disparity that arises between *E*_p_ and *E*^0^. Appropriate thermodynamic values of the disulfide redox couple cannot be directly determined from standard electrochemical measurements owing to the facile bond dissociation of the disulfide radical anion. As we emphasize here, using the transfer coefficient to determine *E*^0^, we show that the disulfide radical anions are generally strong reductants. Indeed, an extremely strong reductant is derived from an amino acid parentage by placing an electron within the strongly reducing environment of the S–S bond of cysteine. The reduction potential of −1.38 V *vs.* NHE makes CysS–SCys˙^−^ one of the strongest reducing agents in biology. Such reducing power has been harnessed by RNR to drive the conversion of RNA building blocks to DNA building blocks, thus underpinning the redox process that supplies the building blocks for DNA synthesis and repair. More generally, the transient nature of CysS–SCys˙^−^ has made it difficult to observe in biological processes. Nonetheless, CysS–SCys˙^−^ may be more prevalent than recognized to date in redox processes in biology, especially for the reaction of substrates requiring exceptionally strong reducing power.

## Data availability

Experimental methods and detail results are provided in the ESI.†

## Author contributions

Q. Z. performed research and contributed new reagents/analytic tools; Q. Z., C. C., J. S. and D. G. N. designed research and analysed data; Q. Z., C. C., J. S. and D. G. N. prepared the manuscript.

## Conflicts of interest

There are no conflicts to declare.

## Supplementary Material

SC-014-D3SC01867A-s001
