# Supplementary material for: Disulfide radical anion as a super-reductant in biology and photoredox chemistry
Source: Chem Sci. 2023 May 17;14(25):6876–81. doi: 10.1039/d3sc01867a (PMC10306091; doi:10.1039/d3sc01867a)
Supplement: SC-014-D3SC01867A-s001 [file SC-014-D3SC01867A-s001.pdf]

Electronic Supplementary Material (ESI) for Chemical Science.
This journal is © The Royal Society of Chemistry 2023

Supporting Information

Disulfide Radical Anion as a Super-Reductant in Biology and Photoredox Chemistry

Qilei Zhu,[a,b,]* Cyrille Costentin,[c] JoAnne Stubbe,[a,d] Daniel G. Nocera[a,]*

[a] Department of Chemistry and Chemical Biology, Harvard University, 12 Oxford Street, Cambridge, MA 02138. [b] Department of Chemistry, University of Utah, Salt Lake City, Utah 84112. Université Grenoble Alpes, CNRS, DCM, 38000 Grenoble, France. [d] Departments of Chemistry and Department of Biology, Massachusetts Institute of Technology, Cambridge, Massachusetts 02139.

Email: dnocera@fas.harvard.edu; q.zhu@utah.edu

Table of Contents

A. Materials and Methods………………………………………………………………………. S3

B. Synthesis and Characterization of Disulfides ……………………………….………………… S3

C. Electrochemistry…………………………….………………………………….……………… S7

Figure S1. Cyclic voltammograms and E_p-α plots of disulfides ……………….……..…... S8

Figure S2. λ vs E_p–E^0 plots for compounds of disulfides ……………….………...…......S11

Figure S3. Cyclic voltammograms of cyclohexanone derivatives……………...……...... S12

Figure S4. Breslow-Bordwell cycle for determining E^0 of furanone……………..….…... S13

D. References……………………………………………………………………….…………… S14

A. Materials and Methods

Compound **1** was purchased from TCI America and compounds **5**, **6**, **7**, **8** were purchased from Sigma-Aldrich. Disulfanediyldiacetic acid and 3,3′-disulfanediyl(2*R*,2′*R*)-bis(2-aminopropanoate) dihydrogen chloride were purchased from Millipore Sigma. All solvents were dried and deoxygenated on a solvent purification system or with three cycles of freeze-pump-thaw and stored over activated 3 Å molecular sieves. Organic solutions were concentrated under reduced pressure on a Büchi rotary evaporator. Chromatographic purification of products was accomplished by flash chromatography on Silicycle F60 silica gel. Thin-layer chromatography (TLC) was performed on Silicycle 250 μm silica gel plates. Visualization of the developed chromatogram was performed by fluorescence quenching, Seebach's stain and potassium permanganate stain.

^{1}H, ^{13}C and ^{19}F NMR spectra were recorded on an Agilent DD2 spectrometer operating at 600 MHz or a Varian Unity/Inova spectrometer operating at 500 MHz or a JEOL ECZ400S spectrometer operating at 400 MHz, both of which are housed in the Harvard University Department of Chemistry and Chemical Biology NMR facility. Chemical shifts were calibrated by internal standard tetramethylsilane (TMS). Data for ^{1}H NMR are reported as: chemical shift (δ ppm), integration, multiplicity (s = singlet, d = doublet, t = triplet, q = quartet, m = multiplet), coupling constant (Hz) and assignment. Data for ^{13}C NMR are reported in terms of chemical shift and no special nomenclature is used for equivalent carbons.

B. Synthesis and Characterization of Disulfides

Compound **2**

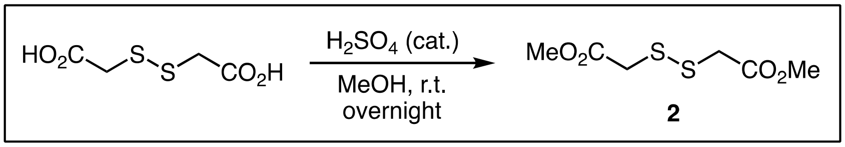

Dimethyl 2,2′-disulfanediyldiacetate (**2**) was synthesized according to a reported procedure.[1] 2,2′-Disulfanediyldiacetic acid (3.6 g, 20 mmol, 1.0 equiv) was added into 100 mL MeOH, five minutes later, 0.5 mL concentrated H_2SO_4 was added. The mixture was stirred at room temperature overnight. After completion, methanol was removed under reduced pressure. The remaining residue was pooled into diethyl ether, the organic layer washed with brine, saturated sodium bicarbonate (aq) and brine. The organic layer was then dried over anhydrous sodium sulfate. The diethyl ether layer was concentrated under reduced pressure. The obtained crude product was purified by silica gel column flash chromatography (hexane to 25% ethyl acetate in hexane). Pure product was obtained as colorless oil, 3.8 g, 90% yield. The NMR characterization data is consistent with previous reports.[1]

^{1}H NMR (400 MHz, chloroform-*d*) δ 3.76 (s, 3H), 3.59 (s, 2H).

^{13}C NMR (101 MHz, chloroform-*d*) δ 170.58, 52.64, 41.21.

Compound **4**

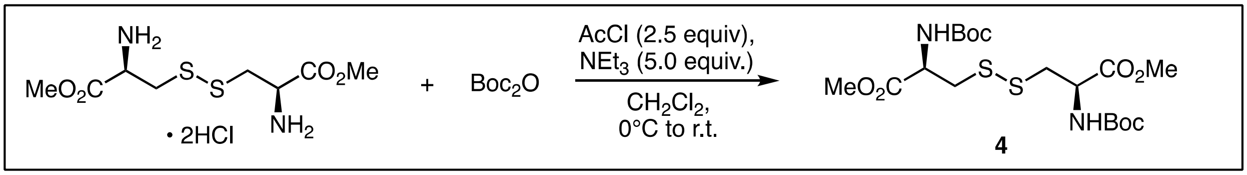

Dimethyl 3,3′-disulfanediyl(2*R*,2'*R*)-bis(2-((*tert*-butoxycarbonyl)amino)propanoate) (**4**) was synthesized according to a reported procedure.[2] A 250 mL round bottom flask charged with a stir bar was added dimethyl 3,3′-disulfanediyl(2*R*,2'*R*)-bis(2-aminopropanoate) dihydrogen chloride (5.12 g, 15 mmol, 1.0 equiv), water (75 mL), tetrahydrofuran (75 mL), 10 mL of triethylamine (7.6 g, 75 mmol, 5.0 equiv), and di-*tert*-butyl dicarbonate (6.9 g, 31.5 mmol, 2.1 equiv) in an ice bath. The reaction mixture was allowed to stir at room temperature for 12 h. After completion, THF was removed under reduced pressure and the resulting aqueous solution was extracted with ethyl acetate (3 × 150 mL). The combined organic layers were washed three times with brine and dried over anhydrous sodium sulfate and concentrated under reduced pressure. The crude product was purified by silica gel column flash chromatography (hexane to 30% ethyl acetate in hexane) to yield a white solid (5.1 g, 73% yield). The NMR characterization data is consistent with previous reports.[3]

^{1}H NMR (400 MHz, chloroform-*d*) δ 5.37 (d, *J* = 6.0 Hz, 2H), 4.59 (d, *J* = 6.6 Hz, 2H), 3.77 (s, 6H), 3.15 (d, *J* = 5.3 Hz, 4H), 1.44 (s, 18H).

^{13}C NMR (101 MHz, chloroform-*d*) δ 171.17, 155.04, 80.31, 52.80, 52.64, 39.85, 27.47.

Compound **9**

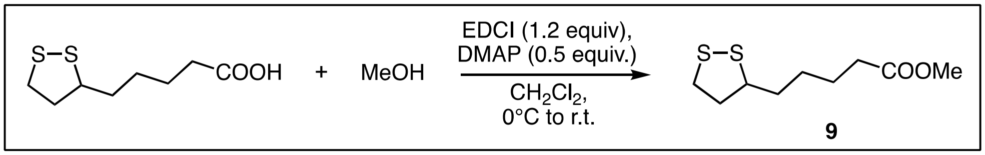

Methyl 5-(1,2-dithiolan-3-yl)pentanoate (**9**) was synthesized according to a published procedure.[4] An oven-dried 250 mL round bottom flask charged with a magnetic stir bar was added anhydrous dichloromethane (100 mL), lipoic acid (6.2 g, 30 mmol, 1.0 equiv), methanol (9.61 g, 10 equiv, 300 mmol), 4-(*N*,*N*-dimethylamino)pyridine (1.8 g, 15.0 mmol, 0.5 equiv), *N*-(3-dimethylamino-propyl)-*N*′-ethylcarbodiimide hydrochloride (EDCI) (6.9 g, 36 mmol, 1.2 equiv) in an ice bath. The reaction mixture was stirred and warmed to room temperature overnight. The suspension was diluted with ethyl acetate and washed with 1 M HCl (aq), brine, saturated $NaHCO_3$ (aq), and brine. The organic phase was separated, dried over anhydrous Na_2SO_4, filtered, and concentrated in vacuo. The crude product was purified by silica gel column flash chromatography (hexane to dichloromethane) to yield the desired product as a yellow oil (5.6 g, 85% yield).

Compound **10**

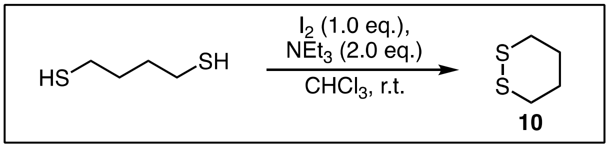

1,2-Dithiane (**10**) was synthesized according to a reported procedure.[5] Butane-1,4-dithiol (2.4 g, 20 mmol, 2.3 mL, 1.0 equiv) was dissolved in $CHCl_3$ (125 mL). The solution was transferred to a dropping funnel on a 500 mL three-necked flask equipped with a magnetic stirring bar. Another dropping funnel was filled with iodine (5.1 g, 20 mmol, 1.0 equiv) dissolved in $CHCl_3$ (150 mL). The two solutions were added dropwise and simultaneously to triethylamine (4.0 g, 40 mmol, 5.8 mL) in $CHCl_3$ (100 mL) over a period of 3 h. After the addition was completed, the resulting light orange solution was stirred for additional 2 h at room temperature. After the reaction was completed, the organic solution was washed with sodium thiosulfate solution, 0.1 M HCl (aq) and brine. The solvent was removed under reduced pressure after drying over anhydrous Na_2SO_4. The resulting pale-yellow oil was further purified using silica gel column flash chromatography using 100% hexane as eluent to give the desired product, as colorless oil (1.9 g, 80% yield). The NMR characterization data is consistent with previous reports.[5]

^{1}H NMR (400 MHz, chloroform-*d*) δ 2.85 (t, J = 4.3 Hz, 2H), 1.97 (dt, J = 5.4, 2.5 Hz, 2H).

^{13}C NMR (101 MHz, chloroform-*d*) δ 33.36, 27.80.

Compound **11**

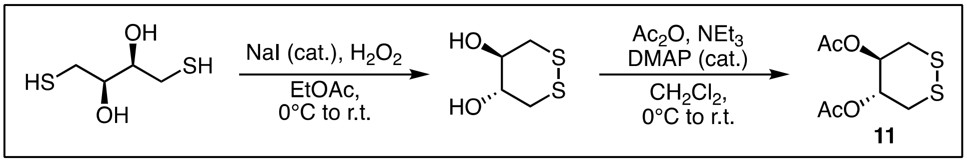

(±)-(4*R*,5*R*)-1,2-Dithiane-4,5-diol (**11**) was synthesized according to a reported procedure.[6] (±)-(2*R*,3*R*)-1,4-Dimercaptobutane-2,3-diol (4.9 g, 30 mmol, 1.0 equiv) was added to a 150 mL round bottom flask equipped with a magnetic stir bar and dissolved in 60 mL ethyl acetate and cooled to 0 °C. Sodium iodide (45 mg, 0.30 mmol, 0.010 equiv) was added to the reaction mixture serving as a catalyst. Hydrogen peroxide (33% w/w; 3.4 mL, 30 mmol, 1.4 equiv) was added dropwise over 30 min. The ice bath was then removed after addition of hydrogen peroxide to allow the reaction to proceed at room temperature for 2 h. The color of the solution changed from colorless to brown as an indication of the completion of the reaction. The reaction was then quenched by addition of saturated sodium thiosulfate. The solution then became colorless after which it was diluted with addition of 60 mL EtOAc, and the aqueous layer was removed using a separatory funnel. The organic layer was then washed with brine and dried over anhydrous sodium sulfate.

The solvent was removed under reduced pressure to yield the crude product as a white powder (2.8 g, 61%). The crude product was subject to the next step without further purification.

(±)-(4*R*,5*R*)-1,2-Dithiane-4,5-diol (3.0 g, 20 mmol, 1.0 equiv) and dichloromethane (40 mL) were added to a flame dried 100 mL round bottom flask charged with a magnetic stirred bar. The solution was cooled in an ice bath, followed by addition of 4-dimethylaminopyridine (0.24 g, 2.0 mmol, 0.1 equiv), and triethylamine (8.1 g, 80 mmol, 11 mL, 4.0 equiv). Acetic anhydride (5.1 g, 50 mmol, 5.1 mL, 2.5 equiv) was added slowly. The solution was warmed to room temperature and stirred overnight. After reaction completion, the reaction was diluted with another 40 mL of dichloromethane, and the organic layer was washed with 1 M HCl (aq), and brine. The organic phase was dried over anhydrous sodium sulfate, followed by concentration under reduced pressure. The residue was purified using silica gel column flash chromatography (10% EtOAc in hexane to 40% EtOAc in hexane) to yield the desired product, as a white solid (4.1 g, 87% yield).

^{1}H NMR (400 MHz, chloroform-*d*) δ 5.10 – 4.93 (m, 1H), 3.16 (dd, *J* = 13.3, 3.0 Hz, 1H), 3.12 – 2.98 (m, 1H), 2.05 (s, 3H).

^{13}C NMR (101 MHz, chloroform-*d*) δ 169.79, 72.08, 38.30, 20.88.

Compound **12**

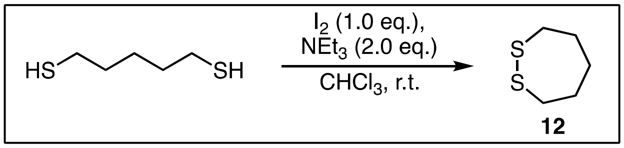

1,2-Dithiepane (**12**) was synthesized according to a reported procedure.[5] Pentane-1,5-dithiol (2.7 mL, 2.7 g, 20 mmol, 1.0 equiv) was dissolved in $CHCl_3$ (125 mL). The solution was transferred to a dropping funnel on a 500 mL three-necked flask equipped with a magnetic stirring bar. Another dropping funnel was filled with iodine (5.1 g, 20 mmol, 1.0 equiv) dissolved in $CHCl_3$ (150 mL). The two solutions were added dropwise and simultaneously to triethylamine (4.0 g, 40 mmol, 5.8 mL) in $CHCl_3$ (100 mL) over a period of 3 h. After the addition was completed, the resulting light orange solution was stirred for additional two hours at room temperature. After the reaction was completed, the organic solution was washed with sodium thiosulfate solution, 0.1 M HCl (aq) and brine. The solvent was removed under reduced pressure after drying over anhydrous Na_2SO_4. The resulting pale-yellow oil was further purified using silica gel column flash chromatography using first 100% hexane as eluent to give the desired product, as colorless oil (2.1 g, 79% yield). The NMR characterization data is consistent with previous reports.

^{1}H NMR (400 MHz, chloroform-*d*) δ 2.83 (d, *J* = 6.0 Hz, 1H), 2.13 – 1.95 (m, 1H), 1.83 – 1.73 (m, 1H).

^{13}C NMR (101 MHz, chloroform-*d*) δ 39.47, 30.25, 26.25.

Compound **14**

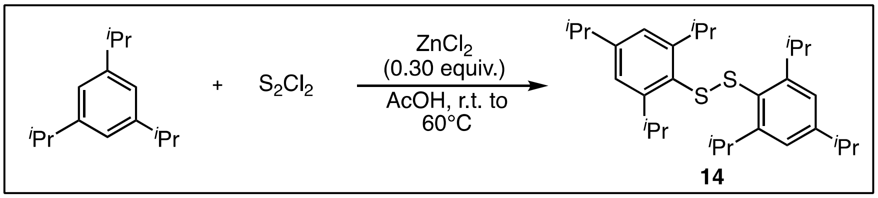

1,2-bis(2,4,6-triisopropylphenyl)disulfane (**14**) was synthesized according to our published procedure.[7]

C. Electrochemistry

General procedure of cyclic voltammetry experiments: 388 mg of tetrabutylammonium hexafluorophosphate ($TBAPF_6$, 0.1 M) was dissolved in 10 mL of anhydrous acetonitrile. The solution was transfer to an oven-dried three-neck flask equipped with a polished 3 mm diameter glassy carbon button working electrode, Ag^+/Ag reference electrode (in acetonitrile) and a platinum mesh counter electrode. The solution was degassed by bubbling Ar for 15 min and was maintained under positive Ar pressure. Background current was measured with scan rates of 0.1, 0.2, 0.5, 1.0, 2.0, 5.0 V/s. Then 3.6 mg disulfide **1** (1.5 mM) was added and the solution was bubbled for another 10 min. One set of cyclic voltammograms were taken with scan rate of 0.1, 1.0, 0.2, 2.0, 0.5, 5.0 V/s, and another set with reverse order to test if systematic degradation/surface modification is happening during the electrochemical experiment. The experiment was repeated twice to calculate the standard deviation. The potential readings were calibrated with 0.5 mM ferrocene solution in acetonitrile. The cyclic voltammetry traces of **1** were presented after subtraction of the non-Faradaic background current and divided by the square root of scan rate.

The symmetry factor/transfer coefficient (α) was determined as described in the main text of the manuscript. E_p-α plots were extrapolated to $\alpha = 0.5$ to furnish $E^0_{RSSR/RSSR\bullet-}$. The CV and E_p-α plot is provided in the main text. The standard reduction potentials of **3**[2] and **13**[8] have previously been reported. CVs and E_p-α plots for **2, 4–12** are provided in Figure S1.

Figure S1

Compound **2**

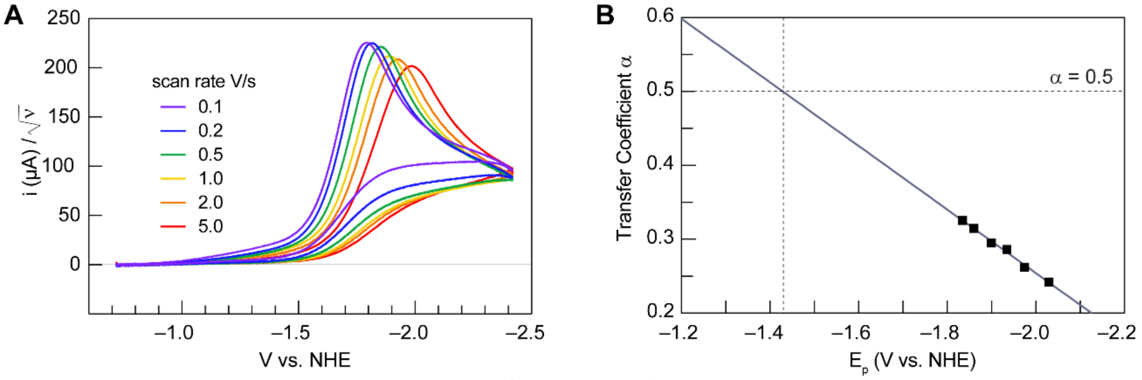

Compound **4**

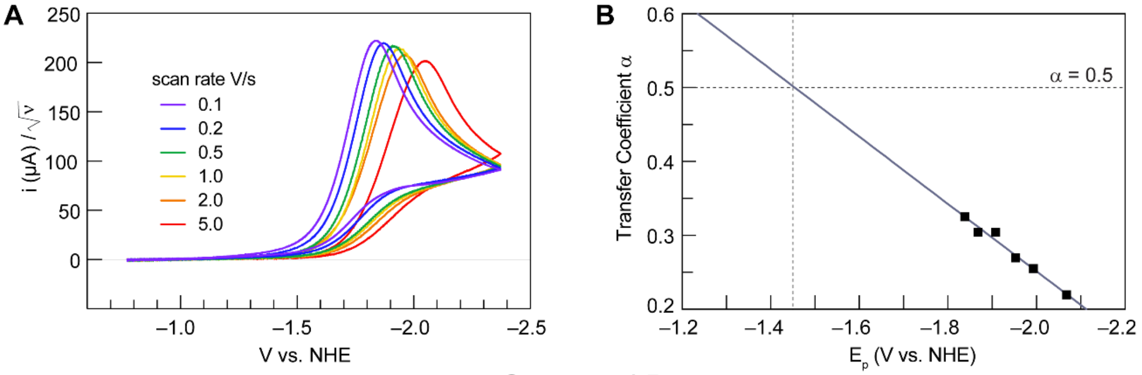

Compound **5**

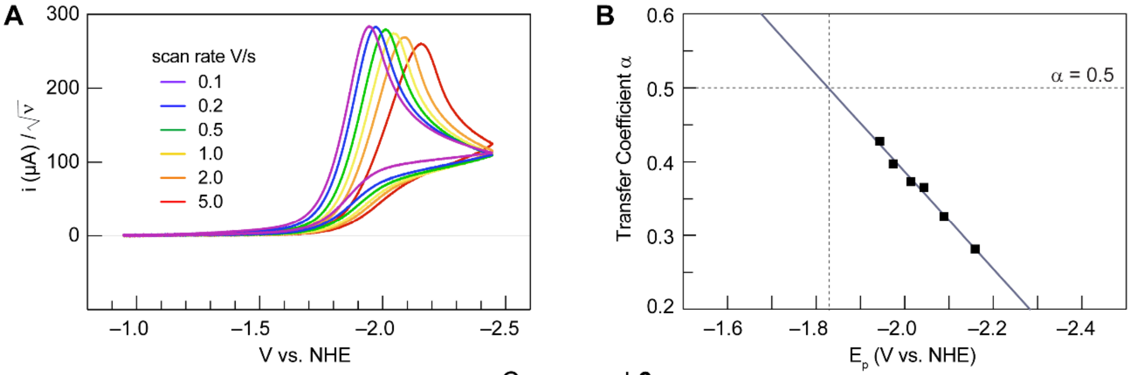

Compound **6**

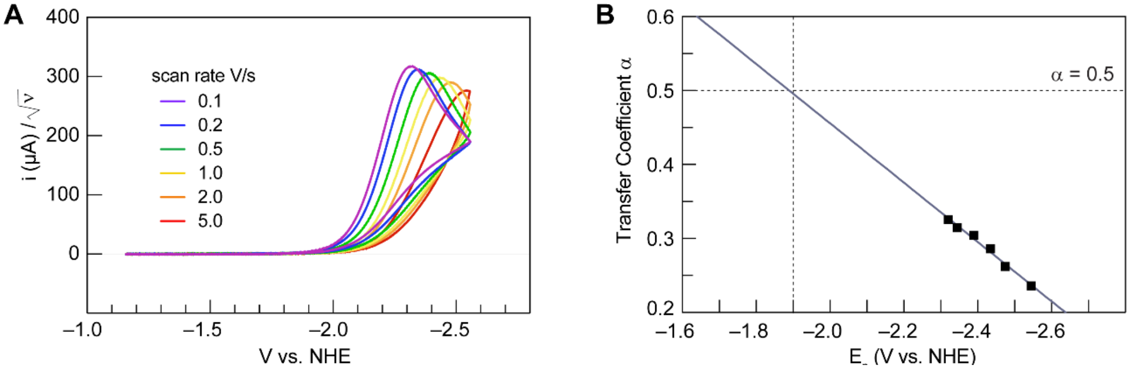

Figure S1 (cont'd)

Compound **7**

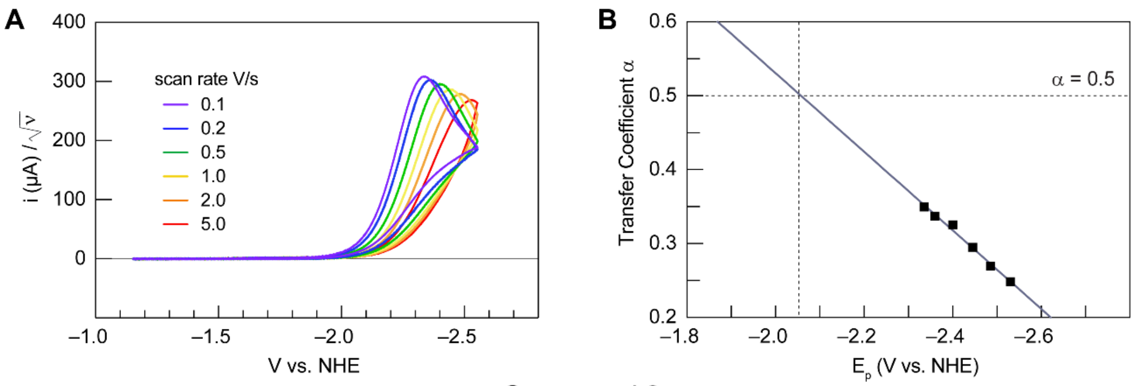

Compound **8**

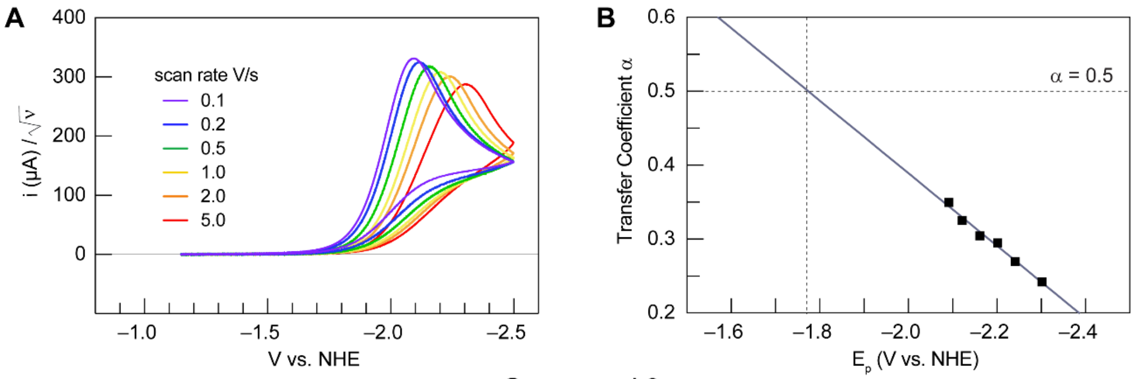

Compound **9**

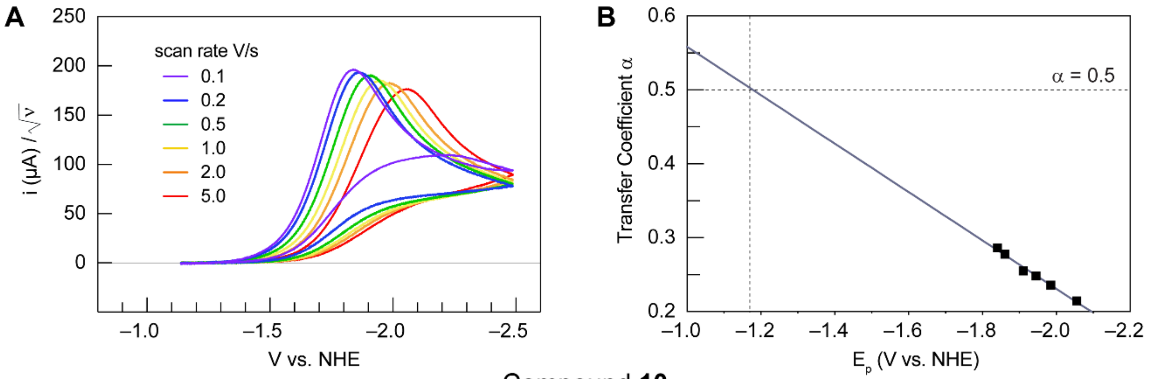

Compound **10**

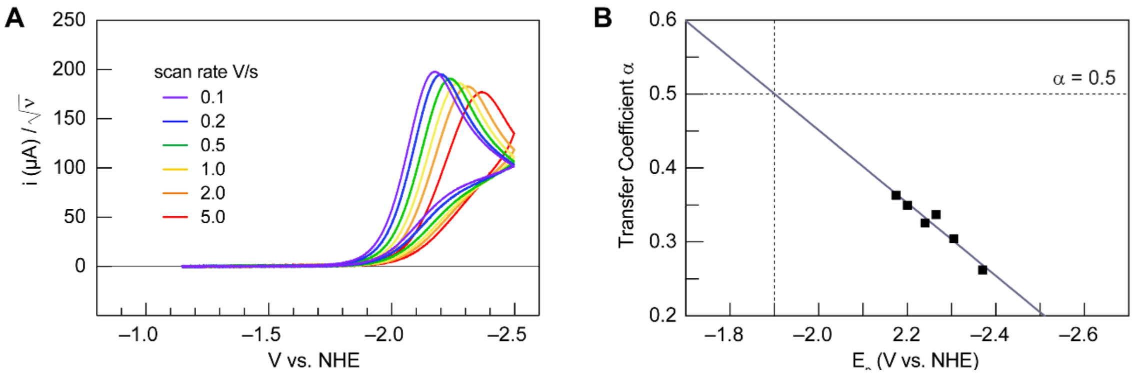

Figure S1 (cont'd)

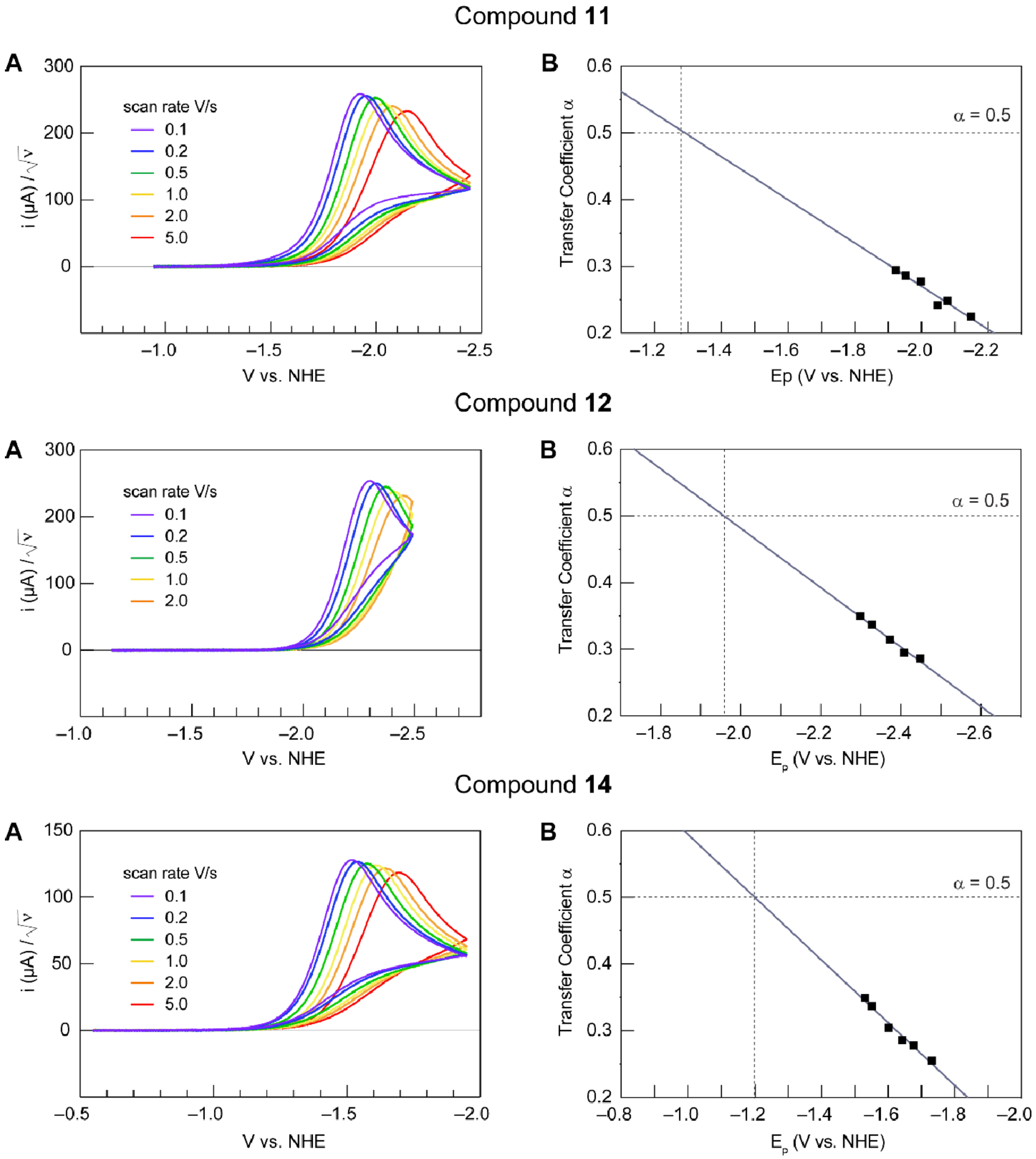

Figure S1. (**A**) Cyclic voltammograms and (**B**) peak potential transfer coefficient (E_p-α) plots of disulfide compounds. Electrolyte is anhydrous acetonitrile solution containing 1.5 mM RSSR and 0.1 M tetrabutylammonium hexafluorophosphate.

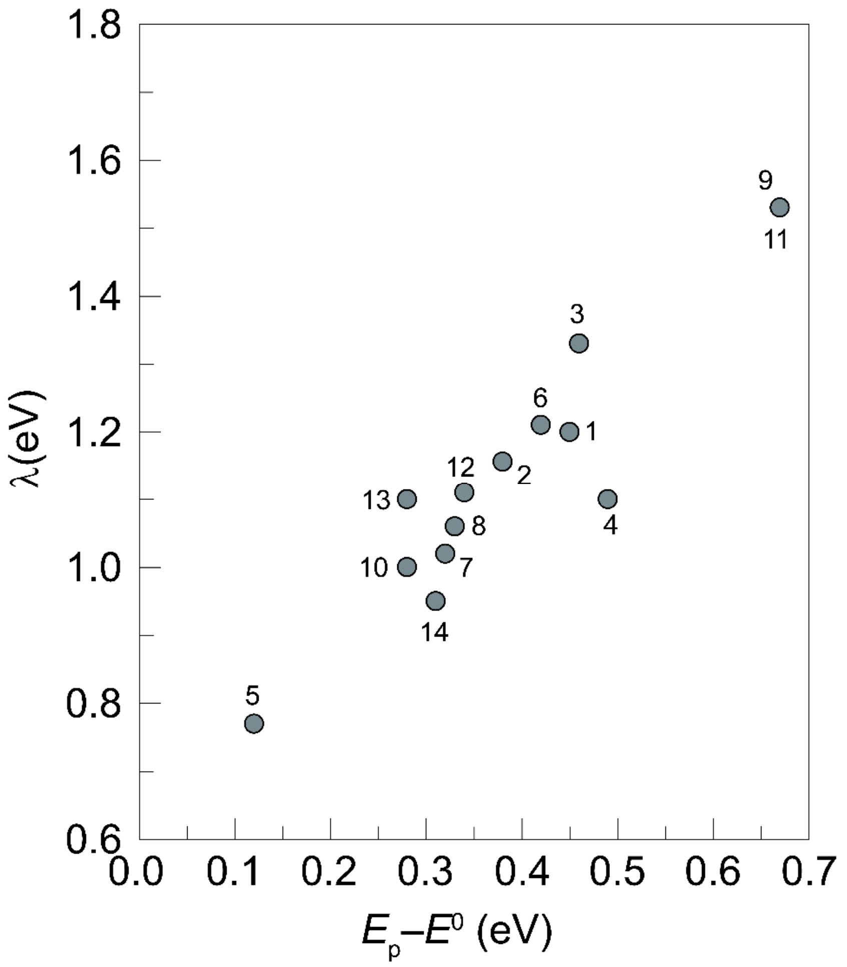

Figure S2. λ vs E_p–E^0 plots for compounds **1**–**14**. λ determined from the slope of E_p-α plots of Figure S1.

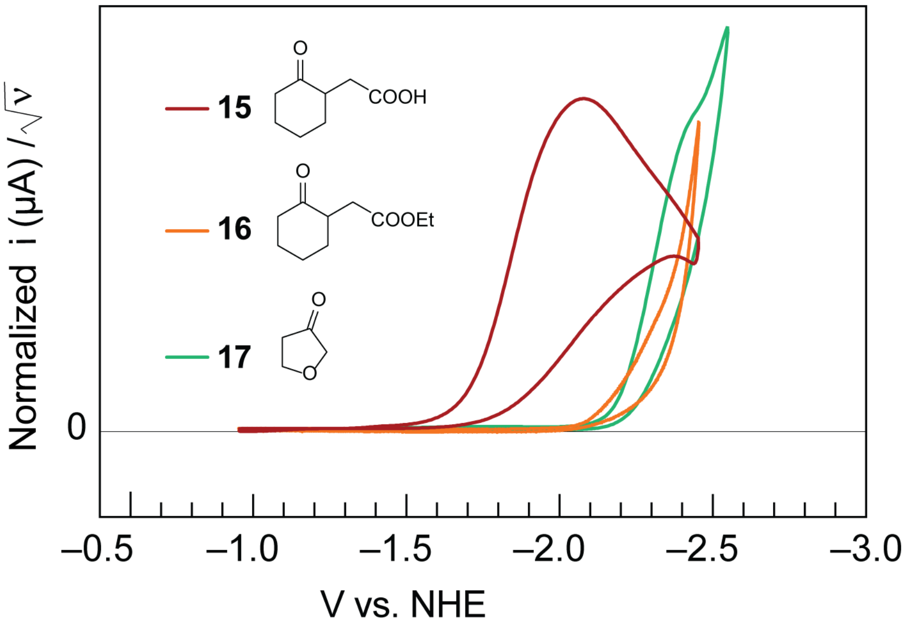

Figure S3. Cyclic voltammograms of cyclohexanone derivatives (1.5 mM), 0.1 V/s in propylene carbonate.

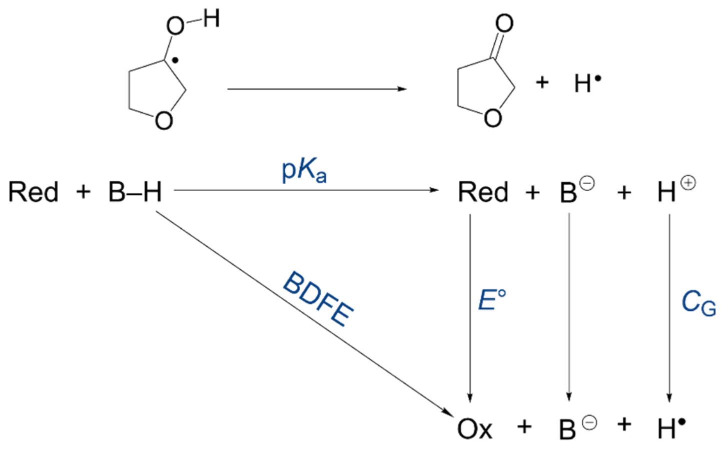

Breslow–Bordwell Equation:

$$\mathrm{BDFE} = 1.37\,\mathrm{p}K_a + 23.06\,E^\circ + C_G$$

Figure S4. Estimation of redox potential for the reduction of furanone using Breslow-Bordwell equation.

D. References

1. X. Lei, Y. Wang, E. Fan, Z. Sun, In Situ Activation of Disulfides for Multicomponent Reactions with Isocyanides and a Broad Range of Nucleophiles. *Org. Lett.* 2019, **21**, 1484–1487.

2. Z. Li, Z. Gentry, B. Murphy, M. S. VanNieuwenhze, Scalable Synthesis of Orthogonally Protected β-Methyllanthionines by Indium(III)-Mediated Ring Opening of Aziridines. *Org. Lett.* 2019, **21**, 2200–2203.

3. P. Mampuys, Y. Zhu, S. Sergeyev, E. Ruijter, R. V. A. Orru, S. V. Doorslaer, B. U. W. Maes, Iodide-Catalyzed Synthesis of Secondary Thiocarbamates from Isocyanides and Thiosulfonates. *Org. Lett.* 2016, **18**, 2808–2811.

4. Y. Liu, Y. Jia, Q. Wu, J. S. Moore, Architecture-Controlled Ring-Opening Polymerization for Dynamic Covalent Poly(disulfide)s. *J. Am. Chem. Soc.* 2019, **141**, 17075–17080.

5. H. Abul-Futouh, L. R. Almazahreh, M. K. Harb, H. Görls, M. El-khateeb, W. Weigand, [FeFe]-Hydrogenase H-Cluster Mimics with Various $-S(CH_2)_nS-$ Linker Lengths (n = 2–8): A Systematic Study. *Inorg. Chem.* 2017, **56**, 10437–10451.

6. S. Pal, A. Sommerfeldt, M. B. Davidsen, M. Hinge, S. U. Pedersen, K. Daasbjerg, Synthesis and Closed-Loop Recycling of Self-Immolative Poly(dithiothreitol). *Macromolecules* 2020, **53**, 4685–4691.

7. Y. Qin, Q. Zhu, R. Sun, J. M. Ganley, R. R. Knowles, D. G. Nocera, Mechanistic Investigation and Optimization of Photoredox Anti-Markovnikov Hydroamination. *J. Am. Chem. Soc.* 2021, **143**, 10232–10242.

8. S. Antonello, R. Benassi, G. Gavioli, F. Taddei, F. Maran, Theoretical and Electrochemical Analysis of Dissociative Electron Transfers Proceeding through Formation of Loose Radical Anion Species: Reduction of Symmetrical and Unsymmetrical Disulfides. *J. Am. Chem. Soc.* 2002, **124**, 7529–7538.
